# Epigenetic change in e-cardherin and COX-2 to predict chronic periodontitis

**DOI:** 10.1186/1479-5876-8-110

**Published:** 2010-11-04

**Authors:** Wings TY Loo, Lijian Jin, Mary NB Cheung, Min Wang, Louis WC Chow

**Affiliations:** 1 Faculty of Dentistry, The University of Hong Kong, Hong Kong, China; 2 Keenlink Dental Clinic, Hong Kong, China; 3 State Key Laboratory for Oral Diseases and Department of Prosthodontics, West China College of Stomatology, Sichuan University, China; 4 UNIMED Medical Institute and Organisation for Oncology and Translational Research Hong Kong, China

## Abstract

**Background:**

DNA methylation of certain genes frequently occurs in neoplastic cells. Although the cause remains unknown, many genes have been identified with such atypical methylation in neoplastic cells. The hypermethylation of E-Cadherin and Cyclooxygenase 2 (COX-2) in chronic inflammation such as chronic periodontitis may demonstrate mild lesion/mutation epigenetic level. This study compares the hypermethylation status of E-Cadherin and COX-2 genes which are often found in breast cancer patients with that in chronic periodontitis.

**Methods:**

Total DNA was extracted from the blood samples of 108 systemically healthy non-periodontitis subjects, and the gingival tissues and blood samples of 110 chronic periodontitis patient as well as neoplastic tissues of 106 breast cancer patients. Methylation-specific PCR for E-Cadherin and COX-2 was performed on these samples and the PCR products were analyzed on 2% agarose gel.

**Results:**

Hypermethylation of E-Cadherin and COX-2 was observed in 38% and 35% of the breast cancer samples, respectively. In chronic periodontitis patients the detection rate was 25% and 19% respectively, and none was found in the systemically healthy non-periodontitis control  subjects. The hypermethylation status was shown to be correlated among the three groups with statistical significance (p < 0.0001). The methylation of CpG islands in E-Cadherin and COX-2 genes in periodontitis patients occurs more frequently in periodontitis patients than in the control subjects, but occurs less frequently than in the breast cancer patients.

**Conclusions:**

This set of data shows that the epigenetic change in E-Cadherin and Cyclooxygenase-2 is associated with chronic periodontitis. The epigenetic changes presented in chronic inflammation patients might demonstrate an irreversible destruction in the tissues or organs similar to the effects of cancer. Chronic periodontitis to some extent might be associated with DNA hypermethylation which is related to cancer risk factors.

## Background

DNA methylation is an epigenetic process that alters DNA chemically. It typically occurs in CpG poor regions, and the promoter region of the gene is not methylated [1]. This process is unsurprisingly occurring and is frequently needed for proper development [2].

Nevertheless, in various types of cancer, including breast ductal carcinoma, abnormal methylation frequently occurs in neoplastic cells. The neoplasia creates a 'methylation imbalance', and causes hypomethylation across the genome and localised hypermethylation within the CpG clusters, or 'islands', in the promoter region of genes that isn't normally methylated [1]. This aberrant methylation in cells causes the silencing of certain genes, including the tumour suppression genes that control DNA repair, cell cycle control, as well as angiogenesis [2]. The cause of such atypical methylation in neoplastic cells is still unknown. CpG island hypermethylation is closely linked to a variety of conditions, including tumorigenesis, chronic inflammation, and intestinal metaplasia [3].

There are many key indicators of cancer, of which two are significant to this investigation: Cyclooxygenase 2 **(**COX-2) and E-Cadherin, as they provide links to chronic periodontitis. COX-2 is, undetectable in most normal tissues, a protein that acts as an enzyme that catalyses the conversion of arachidonic acid into prostaglandins, messengers that promote inflammation [4,5]. Cancer patients have been shown to have overexpression of COX-2 in their malignant tissues [4]. Research has also shown that people who regularly take non-steroidal anti-inflammatory medication such as COX-2 inhibitors had a lower risk of cancer related illnesses, in particular, colorectal cancer and gastric cancer [6]. E-Cadherin is a transmembrane glycoprotein that is responsible for epithelial intercellular adhesion, and is an important gene that regulates morphogenesis [7]. It is also a tumour suppressing gene. Decreased E-Cadherin expression is characteristic of cancer, including lung, prostate, gastric cancers as well as colorectal carcinoma and breast cancer [8].

E-Cadherin and COX-2 expression are useful tools in diagnosing and treating cancer. However, they are also indicators of chronic inflammatory diseases such as periodontitis, as both breast cancer and periodontitis are closely related to CpG island hypermethylation resulting in the silencing of these genes.

Periodontitis is a general term for severe infection of the gums causing inflammation. It can be a result of the worsening of gingivitis [9]. The periodontium, the gums and bones that support the teeth, are destroyed, leading to eventual loss of teeth as well as a possible increased risk of heart attacks and stroke [10,11]. Periodontitis is often chronic and the patient goes through exacerbation and remission periods. It is typically diagnosed via clinical examination, sometimes with the help of x-rays and treatment involves cleaning plaque from the areas under the gums, root scaling and oral antibiotics [12,13]. In advanced stages of the disease, surgery to remove the deep pockets in the gums can be performed [13]. Periodontal disease was significantly associated with cancer risk [14].

COX-2 is obviously related to chronic periodontitis as it is the enzyme responsible for controlling the production of prostaglandins that promote the inflammation characteristic of the disease. It is found in the infected gum tissue of gingivitis and periodontitis patients and therefore COX-2 inhibitors improve the symptoms of periodontitis by reducing the swelling and pain [15]. In addition, studies have shown that the expression of E-Cadherin is uniformly present in healthy gums, but decreased expression of the protein were found in the basal areas of the oral gingival epithelium in diseased samples [9]. This suggests that E-Cadherin expression plays a role in the progression of chronic periodontitis.

This study is to determine the relationship between hypermethylation in chronic periodontitis and breast cancer by comparing the hypermethylation of the E-Cadherin and COX-2 genes in the infected gingival tissues of the periodontitis patients and the neoplastic tissues of cancer patients.

## Materials and methods

### Selection of Control and Patient Samples

This study utilized 108 blood samples from periodontitis-free subjects obtained at random from Hong Kong Red Cross between September 2004 and March 2007 to represent a controlled population. These control cases were matched and compared with disease group of which 39 were female and 69 were male, ranging from 18 to 60 years old (median:45). After blood was taken, these subjects underwent a routine dental examination at Keenlink Dental Clinic, Hong Kong, and was determined to be: free from systemic or chronic disease, current and past non-smokers, have no swelling of the lymph nodes, no temporal mandibular joint disease, no soft tissue abnormalities or severe dental caries, no furcation involvement or generalized gingival recession. An intra-oral soft tissue examination revealed mean probing depth, dental calculus and bleeding on probing (BOP) (Table 1).

**Table 1:** Healthy and periodontitis subjects biographical data.

Parameters	Healthy subjects	Periodontitis Patients
Number of Subjects	108	110
Mean Age ± SD	42.8 ± 9.69	42.9 ± 9.71
Male/Female	69/39	88/22
Mean Pocket Depth (mm)	3.0 ± 0.45	5.5 ± 1.12
Dental Calculus	17.14 +/- 6.85	61.5 +/- 24.63
Probe with Bleeding	13.77 +/- 6.69	76.49 +/- 19.13
Mobility	0-I	III

From 2007 to 2009, a total of 110 periodontitis patients were recruited from West China College of Stomatology, Sichuan University. Among these samples, 37 were female and 73 were male, ranging from 18 to 65 years old (median: 44) (Table 1). Their mean pocket depth was 5.5 mm (Table 1). They have been suffering from periodontal disease for over 5 years and have received scaling and root planning every 6 months. Teeth which showed third degree mobility were extracted. Blood was taken from these patients and associated periodontal tissues were cut during tooth extraction for DNA extraction.

A total of 106 pathologically confirmed breast cancer specimens with an average age of 56.2 were obtained at Huaxi Hospital of Sichuan University between 2007 and 2009 (Table 2). All patients were diagnosed with invasive ductal carcinoma. They were administered with 4 cycles of 600 mg/m2 5-fluorouracil, 80 mg/m2 epirubicin and 600 mg/m2 cyclophosphamide at a 3-week interval between each cycle after having had modified radical mastectomy performed.

**Table 2:** Clinical characteristics of 106 breast cancer patients from Sichuan University.

	Total number of patients (N) = 106
**Mean age**	56.2 (range: 26-85)
**Tumor Size**	
≤ 2 cm (T1)	54
2-5 cm (T2)	35
>5 cm (T3)	17
**Histology**	
Ductal	92
Lobular	14
**Grading**	
G1 and G2	84
G3	22

Written informed consents were obtained from all participants before the procedure which had been approved by ethics committees of the University of Hong Kong and Sichuan University (reference number: 2007SGY028).

### Preparation of Control and Patient Blood Samples

Ten milliliters of blood from each patient was collected in lithium heparin tubes (Vacuette, Austria). The blood was centrifuged for 10 minutes at 1500 rpm and plasma was then removed. The cell pellet was transferred to a 50 ml centrifuge tube and red blood cell lysis buffer was added to a final volume of 45 ml. The mixture in the tube was inverted several times and centrifuged for 10 minutes at 1500 rpm. The supernatant was discarded and the cell pellet was washed with 0.9% PBS to be used for DNA extraction.

### Sample Preparation and Tissue Collection

DNA from the healthy subjects' blood cells, periodontitis patients' tissues and breast cancer tumour samples were extracted by Geneaid^® ^DNA Mini Kit (Tissue) (Geneaid, Taiwan). The provided Micropestle was used to grind the tissue to a pulp. 200 μl of GT Buffer was added into the tube and the sample tissue was continually homogenized with grinding. 20 μl of Proteinase K (10 mg/ml) was added to the sample mixture and mixed by vortexing. The sample was lysed by incubation at 60°C for 30 minutes, and the tube was inverted every 5 minutes. At this time, the required Elution Buffer was preheated at 70°C. 200 μl of ethanol was added to the sample lysate and mixed immediately by vortexing for 10 seconds. Pipetting was performed to break up the precipitate formed. A GD Column was placed in a 2 ml Collection Tube. All the mixture from previous step (including any precipitate) was applied to the GD column, and was centrifuged at 13,000 rpm for 2 minutes. The Collection Tube containing the flow-through was discarded and the GD column was transferred in a new 2 ml Collection Tube. 500 μl of Wash Buffer (ethanol added) was added into the column and was centrifuged at 13,000 rpm for 30 seconds. The flow-through was discarded and the GD column was placed back into the Collection Tube. The wash step by adding Wash Buffer was performed once again. The flow-through was, again, discarded and the GD Column was placed back to the Collection Tube and was centrifuged at full speed for 3 minutes to dry the column matrix. The dry GD Column was transferred in a clean 1.5 ml microcentrifuge tube. 100 μl of preheated Elution Buffer was added into the centre of the column matrix and was stood for 5 minutes until the Elution Buffer was absorbed by the matrix. 13,000 rpm centrifugation was performed for 30 seconds to elute purified DNA.

### Methylation-Specific PCR

The extracted DNA was modified by CpG DNA Modification Kit (CHEMICON INTERNATIONAL, USA). The specific hypermethylated primers for each gene were used for PCR. The sense and antisense primers for the hypermethylated E-Cadherin and COX-2 are listed in Table 3. The PCR mixture consisted of 1× PCR buffer [20 mM Tris-HCl (pH 8.4), 50 mM KCl], 1.5 mM MgCl2, 0.2 mM dNTPs, 40 pmol sense and antisense primers, and 0.75 units of Taq DNA polymerase. Initial denaturation at 94°C for 5 min was followed by 50 cycles of denaturation at 94°C for 30 sec, annealing at 57°C for both hypermethylated and unmethylated sequences for 30 sec and extension at 72°C for 30 sec, and a final extension at 72°C for 10 min. Products of M for E-Cadherin and COX-2 are 115 bp and 116 bp, respectively. The PCR products were analyzed on 2% agarose gel stained with ethidium bromide.

**Table 3:** Primers used and their corresponding sequences

Primer name	Primer's Direction	Primer Sequence
β-actin Gene	Forward	5'-CCACGAAACTACCTTCAACTCC-3'
	Reverse	5'-TCATACTCCTGCTGCTTGCTGATCC-3'
ECAD	Forward	5'-TTAGGTTAGAGGGTTATCGCGT-3'
	Reverse	5'-TAACTAAAAATTCACCTACCGAC-3'
COX-2	Forward	5'-TTAGATACGGCGGCGGCGGC-3'
	Reverse	5'-TCTTTACCCGAACGCTTCCG-3'

### Statistical Analysis

The relative risk of hypermethylation status of E-Cadherin and COX-2 among cancer patients, periodontitis patients and healthy subjects was analysed. Chi-square test was performed to analyse the distribution of hypermethylation in test groups compared with control using SPSS 12.0 (SPSS Inc., USA).

## Results

The methylation specific PCR showed that hypermethylation of the E-Cadherin and COX-2 genes occurred in 38% and 35% of breast cancer patients, respectively. In periodontitis patients, the frequency of hypermethylation of E-Cadherin and COX-2 was 25% and 19%, respectively. However, hypermethylation was not observed in the control group (Table 4). Pearson's chi-square test demonstrated a statistical significance between control group and periodontitis patients for the hypermethylation status of both genes tested (p < 0.0001). Cancer group also showed a statistical significance with the control group (Table 4). The relative risk of periodontitis associated with E-Cadherin and COX-2 was 0.1091(95% confidence interval: 0.005-0.2627) and 0.0485(95% confidence interval: 0.0066-0.3543), respectively.

**Table 4:** Status of E-Cadherin and COX-2 hypermethylation in healthy, periodontitis patients and breast cancer patients.

Groups	Hypermethylation of Cox-2(%)	Chi-square Pearson	Relative Risk (95% confidence Intervals)	Hypermethylation of E-cadherin(%)	Chi-square Pearson	Relative Risk (95% confidence Intervals)
Healthy subjects	0/108(0%)	---	---	0/108(0%)	----	---
(N = 108)						
Periodontitis patients	21/110(19%)	19.82	0.0485	28/110(25%)	28.43	0.1091
(N = 110)		p < 0.0001	0.0066-0.3543		p < 0.0001	0.005-0.2627
Breast cancer patients	37/106(35%)	42.29	0.0265	40/106(38%)	46.8	0.0245
(N = 106)		p < 0.0001	0.0037-0.1899		p < 0.0001	0.0034-0.1753

100 bp DNA marker was selected as a reference. The products of the PCR generated by methylation-specific PCR were used in electrophoresis, using a 100 bp ladder (Invitrogen, USA) and the images were captured under UV light. The hypermethylation status of E-Cadherin and COX-2 was shown to be correlated between the three groups with statistical significance (p < 0.0001).

## Discussion

There are a number of other widely accepted factors contributing to a patient's relative risk to periodontitis. These include age, gender, oral hygiene, smoking, poor glycemic control in diabetic patients, genetics, systemic diseases, etc [16-22]. Apart from these factors, there is also the epigenetic factor. However, the impact of epigenetics on periodontitis and other chronic inflammatory diseases is not studied in as much depth as the other host and genetic factors. Some research has been done on aberrant CpG hypermethylation in other chronic inflammatory diseases such as gastritis and ulcerative colitis but next to none has been done on periodontitis [23,3].

Currently, the diagnosing of chronic periodontitis relies on clinical inspection via probing depths, attachment levels, bleeding, plaque index and the use of x-rays or other radiographic methods [24]. The oral cavity of the patient is usually initially inspected for the disease status. Casts, photos and often X-rays are collected for interpretation. However, diagnoses of the disease by monitoring epigenetic changes such as E-Cadherin and COX-2 expression are very rarely used. Although only E-Cadherin and COX-2 were studied in this investigation, a wide variety of other epigenetic changes may be factors involved in the progression of chronic inflammation diseases like periodontitis, and these could prove to be valuable tools in the diagnosis of such diseases. Furthermore, the potential applications of these changes are not limited to diagnostic purposes. Studying epigenetic changes and their relationships with chronic inflammation may provide not only new diagnostic methods, but it could also be useful in developing new treatments.

Periodontal pathogens may induce chronic inflammation and inflammatory responses. These responses may promote carcinogenesis and disrupt the cell cycle [25]. Both genetic and other factors, such as environmental, epigenetic factors may cause chronic inflammatory diseases [26]. A good example is the different genotypes caused by single nucleotide polymorphisms (SNPs) of the pro-inflammatory genes [27], such as interleukin-1 (IL-1), IL-6, and neutrophil. It seems that genetic factors of the host may decide which bacteria to colonize the host, different gene polymorphisms increase the growth of specific bacteria [28].

Epigenetics may be related to tumourigenesis [29], and other diseases such as cardiovascular diseases. A positive link between chronic inflammation and cancer has been published although the progression mechanism is still debated. Increased DNA methylation has been found in chronic inflammation such as chronic gastritis [30], ulcerative colitis [31] as well as in prostate cancers [32].

IL-6 is produced at the inflammation site, which regulates the transition of neutrophils to macrophages, and it helps the stimulation of T and B cells. Its high level has been found in different infections and cancers. Treatment which targets IL-6 and its signalling may prevent chronic inflammatory diseases [33]. DNA methyltransferase (DNMT1) maintains the methylation pattern, when the IL-6 level is low, the p53 promoter region is modified by DNMT-1 and thus p53 expression decreases. The disrupted expression of this tumour suppressor gene plays a key role in cancer initiation [34]. Although the effect of IL-6 to cancer is still unknown, this cytokine may provide a link from bacterial infection to inflammation and cancer. Changes and damages in cells and tissues during inflammation may initiate cancer development. Specific markers of inflammation can be studied to look at the association with increased methylation, so that the underlying mechanisms between chronic inflammation and cancer can be revealed.

The results of this experiment show that methylation of CpG islands in the E-Cadherin and COX-2 genes in periodontitis patients occurs more frequently than in the healthy control group, but less frequently than in the breast cancer patients as supported by Pearson chi-square test (Table 4). The difference in the percentage frequency of hypermethylation for both genes between the healthy subjects and chronic periodontitis patients is statistically significant (p < 0.0001). The finding showed that chronic inflammation and cancer may share the same pattern of genomic and epigenetic changes. Methylation contributes to chronic inflammation, periodontal disease might be a marker of a susceptible immune system or might directly affect cancer risk and it may occur through possible biologic mechanisms [14,35]. The biological pathways may involve epigenetic changes, this altered CpG region presented in chronic inflammation patients, demonstrates an irreversible destruction in the tissues or organs similar to the cancer effects.

## Conclusions

These data confirms that E-Cadherin and COX-2 expression are factors related to periodontitis. Further study of similar epigenetic changes may prove to be extremely useful in the diagnosis and treatment of chronic periodontitis in the future.

## Declaration of competing interests

We declare that we have no financial and personal relationships with other people or organizations that can inappropriately influence our work, there is no professional or other personal interest of any nature or kind in any product, service and/or company that could be construed as influencing the position presented in, the article entitled, ''Epigenetic change in E-Cardherin and COX-2 to predict chronic periodontitis".

## Authors' contributions

WTYL participated in the design of the study and carried out sample preparation and PCR for healthy subjects and breast cancer patients. LJJ participated in the design of the study and performed the statistical analysis. MW helped to draft the manuscript and collected patients' data. MNBC carried sample preparation and PCR for periodontitis patients. LWCC participated in the design of the study and finalised manuscript. All authors read and approved the final manuscript.
